# Quantum tunneling theory of Cooper pairs as bosonic particles

**DOI:** 10.1038/s41598-021-88228-1

**Published:** 2021-04-27

**Authors:** Edgar J. Patiño, Daniel Lozano-Gómez

**Affiliations:** 1 Superconductivity and Nanodevices Laboratory, Departamento de Física, Universidad de los Andes, Carrera 1 No. 18A-12, A.A. 4976-12340, Bogotá, Colombia; 2 School of Physical Sciences and Nanotechnology, Yachay Tech University, Urcuquí, Ecuador; 3 Department of Physics and Astronomy, University of Waterloo, Waterloo, ON N2L 3G1 Canada

**Keywords:** Bose-Einstein condensates, Physics, Condensed-matter physics, Superconducting properties and materials

## Abstract

We propose a simple phenomenological theory for quantum tunneling of Cooper pairs, in superconductor/insulator/superconductor tunnel junctions, for a regime where the system can be modeled as bosonic particles. Indeed, provided there is an absence of quasiparticle excitations (fermions), our model reveals a rapid increase in tunneling current, around zero bias voltage, which rapidly saturates. This manifests as a zero bias conductance peak that strongly depends on the superconductors temperature in a non-monotonic way. This low energy tunneling of Cooper pairs could serve as an alternative explanation for a number of tunneling experiments where zero bias conductance peak has been observed.

## Introduction

Quantum tunneling has been a subject of intensive research since the middle of the twentieth century after its original proposal in quantum mechanics. This phenomenon has been widely investigated both in theory and experiments in a number of branches of physics including; atomic physics for explaining the decay of a nucleus^1^, cosmological physics for study of thermal emission black holes^2,3^, Rb atoms Bose–Einstein (BE) condensates in bosonic Josephson junctions^4–6^, quantum optics in photonic and polaritonic systems^7,8^ and solid-state physics^9–11^ where artificially fabricated tunnel devices have been experimentally realized with multiple applications. Indeed, when two electrodes are separated by a thin insulating material, single electrons can tunnel across the barrier as demonstrated by numerous experiments even at room temperature^10,11^. One of the most popular models for tunnel junctions was proposed by Simmons in 1963^12^, where tunneling of fermionic currents through a junction is described as a function of temperature. This model has been widely used to explain results obtained from quantum tunneling devices and is the inspiration for the work we present here. On the other hand, for superconductors, paired electrons forming Cooper pairs in superconductor/insulator/superconductor (S/I/S) junctions, were predicted to show tunneling effects by Josephson in 1962^13^ and later confirmed experimentally soon after. Indeed, the DC Josephson effect predicted a DC current at zero bias voltage and has been confirmed by an enormous amount of experiments. However, when the voltage is increased at T = 0, no current can flow from one electrode to the other until it reaches ${\mathrm{eV}} = 2\Delta$, the energy needed to break Cooper pairs that leads to quasiparticle current. This is not the case when one of the layers is a normal metal in N/I/S junctions at finite temperatures. The energy of quasiparticles excitation, from the normal metal, allows tunneling at lower voltages via the so-called Andreev reflection processes, not relevant for the present analysis. For S/I/S junctions at finite temperatures when phonon energies and voltages are greater than $2\Delta$, a fraction of Cooper pairs break into quasiparticles producing so-called subgap or excess currents. Given that quasiparticles are made of electrons, tunneling processes are of fermionic nature leading to tunneling current-voltage characteristics of similar shape to the ones obtained for normal tunneling junctions^10^, i.e. an indefinitely increasing function of voltage without saturation value. However, these currents have been experimentally observed at temperatures and voltages below $2\Delta$^14,15^, lower than the energy required for thermally induced excess currents without a clear explanation on its origin. In other experiments, on Nb-based S/I/S junctions^16,17^, considerably higher currents than anticipated from BCS theory were observed, this effect was attributed to the existence of a normal conducting layer on the superconducting film^18,19^. Moreover, a zero-bias conductance peak was observed in S-I-S junctions^20^ by tuning the barrier thickness. The results in Ref.^20^ were also explained by normal layer formation leading to Andreev reflection processes. However in multiple experiments^16–20^ there was no direct evidence of this normal layer. More recently the observation of large currents in Nb-AlO$_{x}$-Nb Josephson junctions arrays were ascribed to experimental evidence of nonuniform boson distribution^21–23^. This was attributed to Bose–Einstein condensation and boson hopping^22,23^ between superconducting islands of the array. In this work, based on the assumption of Cooper pairs behaving as bosons, we derive a theory for the current flow of bosons across a S/I/S tunnel junction. Our theory provides a different mechanism to transfer Cooper pairs as boson-like particles, in addition to quasiparticle tunneling. Our predictions could be observed in experimental setups with voltages and temperatures below the energy gap where small excess currents appear although quasiparticle excitations are absent. Once the voltage and temperature reach $\sim 2\Delta$, boson currents should be completely replaced by quasiparticle currents.

## Tunneling of bosons

Previously, currents in photonic and polaritonic systems, have been studied for massless and chargeless bosons^7,8^ hence voltage has not been included in such models. In contrast, in the present work we consider two boson reservoirs of charged massive particles separated by an insulating barrier. In each reservoir, bosons are assumed to have intrinsic mass ($m^*$) and charge ($e^*$), and share the same energy state at the chemical potential $\mu$ with a probability density given by the Bose–Einstein distribution B(E). The probability $D(E_x)$ that an incident boson, with kinetic energy $E_{x}=m v_{x}^{2}/2$ component along the *x* direction, crosses the potential barrier *V*(*x*) can be described by means of the WKB approximation

1 $$\begin{aligned} D(E_x)=\exp \left( -2\int _{x_1}^{x_2}\sqrt{ \frac{2m*}{\hbar ^2}\left[ V(x)-E_x\right] } dx\right) , \end{aligned}$$

where bosons are subject to a potential energy $V(x)=\mu +\phi (x)$ dictated by the barrier height $\phi (x)$, defined in the interval $[x_1,x_2]$, and chemical potential $\mu$, close to zero at low temperatures. With this description one may calculate the number of bosons tunneling from the left side (N$_1$) and right side (N$_2$) when a potential eV is applied on the right reservoir;

2 $$\begin{aligned} N_1= & {} \frac{4\pi m^{*}}{h^3}\int _0^{E_m} D(E_x)\left[ \int _0^\infty B(E)dE_r\right] dE_x \nonumber \\ N_2= & {} \frac{4\pi m^{*}}{h^3}\int _0^{E_m} D(E_x)\left[ \int _0^\infty B(E+eV)dE_r\right] dE_x, \end{aligned}$$

where $E_m$ is the maximum energy of the electrons and the integral has been expressed in polar coordinates, i.e. $E_r = E_y + E_z$. Altogether, a net bosonic current flow (J$_B$) is obtained from the difference between these two values;

3 $$\begin{aligned} J_B=e^*(N_1-N_2) \end{aligned}$$

which can be rewritten as;

4 $$\begin{aligned} J_B= & {} \frac{4\pi m^{*}e^*}{h^3}\int _0^{E_m}D(E_x)\left[ \int _0^\infty B(E)dE_r-B(E+eV)dE_r\right] dE_x. \end{aligned}$$

In the present work, we consider a fixed rectangular potential barrier $\phi _0$ where the barrier height is constant for the studied voltage regime. This assumption is consistent with the low voltage approximation ($V\approx 0$ as in^12^) where barrier shape does not change upon applied voltage. This is justified when considering superconductors as bosons reservoirs as we shall see in the next section.

Therefore, the potential takes a constant value of $V (x)=\mu +\phi _0$, $\mu$ taken as the highest occupied energy level and $\phi _0$ the barrier height. For the system considered the tunneling probability simplifies to;

5 $$\begin{aligned} D(E_x)= & {} \exp \left( -2\int _{x_1}^{x_2}\sqrt{ \frac{2m*}{\hbar ^2}\left[ \phi _0+\mu -E_x\right] } dx\right) . \end{aligned}$$

The integral in Eq. (5) is further simplified using the approximation used by^12^ obtaining

6 $$\begin{aligned} D(E_x)\approx & {} \exp \left( \frac{-2(2m^*)^{1/2}}{\hbar }\beta s\left[ \mu +\phi _0-E_x\right] ^{1/2} \right) , \end{aligned}$$

where $s=x_2-x_1$ corresponds to the barrier width and $\beta$ is a correction factor which can be chosen to be unity for the low voltage regime^12^. Finally, the temperature dependence is included in the difference of BE distributions integral (BEI) that solved^24^ gives;

7 $$\begin{aligned} {\mathrm {BEI}}= & {} \left[ \int _0^\infty B(E)dE_r-B(E+eV)dE_r\right] = \int _0^{\infty } \left[ \frac{1}{e^{(E-\mu )/k_B T}-1}-\frac{1}{e^{(E+eV-\mu )/k_B T}-1}\right] dE_r \nonumber \\= & {} k_B T\ln \left[ \frac{1-e^{(\mu -E_x-eV)/k_B T} }{1-e^{(\mu -E_x)/k_B T}}\right] , \end{aligned}$$

Altogether, the final relation between current density and voltage across the leads;

8 $$\begin{aligned} J_B= & {} \frac{4\pi m^{*}e^*k_BT}{h^3} \int _0^{E_m} \exp \left( \frac{-2(2m^*)^{1/2}}{\hbar }\beta s\left[ \mu +\phi _0-E_x\right] ^{1/2} \right) \ln \left[ \frac{1-e^{(\mu -E_x-eV)/k_B T} }{1-e^{(\mu -E_x)/k_B T}}\right] dE_x. \end{aligned}$$

## Tunneling of Cooper pairs as bosonic particles: the superconductor case

**Figure 1:**
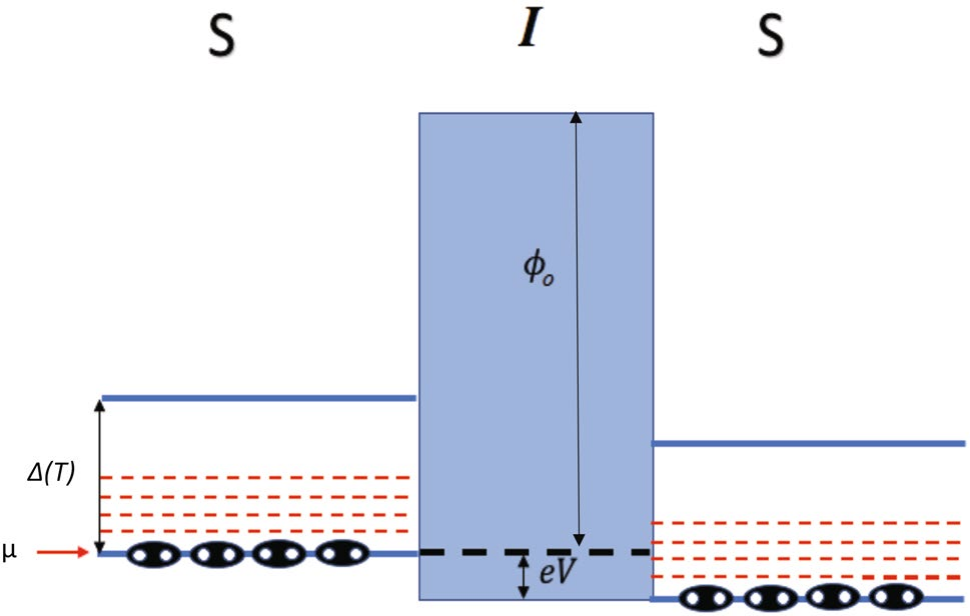
Insulating potential barrier between two superconductors (boson reservoirs). Here Cooper pairs remain as bound particles in the ground state at energies bellow the energy gap $\Delta (T)$ and barrier height $\phi _{o}$.

Consider a superconducting junction where Cooper pairs are assumed to behave as bosons^25–28^ , with a negligible interaction between them and thus obey Bose–Einstein statistics (this is possible for a system of even fermions with finite center of mass momentum^26,27^). For this system, Cooper pairs populate a single energy level $\mu$ at $T=0$, corresponding to the ground state energy of the system. This energy level has the highest density of states and is separated from the quasiparticle energy states by the energy gap $\Delta _0$. On the other hand, for non zero temperature, the BE distribution will allow higher energy states to be occupied by the bosons (Cooper pairs) in the vicinity of the ground state. In this scenario, illustrated in Fig. 1, each electrode can be considered as a Cooper pair/boson reservoir separated by an insulator modeled as a potential barrier. Moreover, due to their superconducting properties, the number of bosons in each reservoir depends on temperature. This dependence leads to a reduction of Cooper pair density with increasing temperature up to $T_{c}$ where it drops to zero. We start from Eq. (6) where we consider a rectangular potential barrier of height $\phi _{0}$ and width *s* valid for a low voltage regime where $eV< \Delta _0 \ll \phi _{0}$. This consideration is required to prevent Cooper pairs from breaking. Furthermore, the fact that $\Delta _0$ is typically of the order of Milli-electron volts while the barrier height $\phi _{0}$ of the order of eV, justifies the assumption of a constant barrier height. For simplicity, we consider $\mu =0$, the correction factor ($\beta =1$), $e^*= 2e$ and $m^*= 2m$ due to the intrinsic nature of bosons in our condensate made up of paired electrons. Applying these considerations, the tunneling probability (Eq. 6) and current density (Eq. 8) can be expressed as

9 $$\begin{aligned} D(E_x)= & {} \exp \left( \frac{-2(4m\phi _0)^{1/2}}{\hbar } s \left[ 1-\frac{E_x}{\phi _0}\right] ^{1/2} \right) \end{aligned}$$

10 $$\begin{aligned} J_B= & {} \frac{16\pi me k_BT }{h^3} \int _0^{E_m} \exp \left( \frac{-2(4m\phi _0)^{1/2}}{\hbar } s \left[ 1-\frac{E_x}{\phi _0}\right] ^{1/2} \right) \ln \left[ \frac{1-e^{(-E_x-eV)/k_B T} }{1-e^{-E_x/k_B T}}\right] dE_x. \end{aligned}$$

Finally, introducing the constant the constant terms $A = 2(4m)^{1/2}\hbar ^{-1} s$, $C_1=\left( \frac{16\pi m e k_B^2T^2}{h^3 }\right) \exp \left( -A\phi _0^{1/2}\right)$, using the binomial approximation in the transmission coefficient in Eq. (10) and the substitution; $u=E_x/k_BT$ the expression for the bosonic current is simplified to:

$$\begin{aligned} J_B= & {} C_1 \int _0^{\frac{Em}{k_BT}} \exp \left( k_B T\frac{A}{2\phi _0^{1/2} } u \right) \ln \left[ \frac{1-e^{-u-(eV/k_BT) } }{1- e^{-u}}\right] du. \end{aligned}$$

This equation is similar to the one found by Simmons^29^, with a different constant $C_1$, potential used and signs in the logarithm. We know that the maximum energy $E_m$ is much greater than the thermal energy that the system can reach thus $E_m /k_BT\gg 1$, this condition let us make an extension to the integral such that;

$$\begin{aligned} J=\frac{C_1}{k_BT} \int _0^{\infty } \exp \left( \frac{A}{2\phi _0^{1/2} }E_x \right) \ln \left[ \frac{1-e^{(-E_x-eV)/k_B T }}{1- e^{-E_x/k_BT}}\right] dE_x. \end{aligned}$$

Using the constants

11 $$\begin{aligned} C_1= & {} \left( \frac{16\pi m e k_B^2T^2}{h^3 }\right) \exp \left( -A\phi _0^{1/2}\right) \nonumber \\ C_2= & {} k_BT\frac{A}{2\phi _0^{1/2} }\nonumber \\ C_3= & {} e^{-eV/k_BT }, \end{aligned}$$

a new equation is obtained:

12 $$\begin{aligned} J= & {} C_1 \int _0^{\infty } \exp \left( C_2 u)\right) \ln \left[ \frac{1-C_3 e^{-u} }{1- e^{-u}}\right] du. \end{aligned}$$

This integral may not have an analytical solution; therefore, one may benefit of the approximation used in Ref.^30^ to simplify the logarithm for currents caused by thermo-ionic effects. The approximation in^30^ can be applied when the energy is greater than the chemical potential, $\mu$, plus a small contribution of $k_B T$. Using this approximation an expression for the current is obtained;

13 $$\begin{aligned} J\simeq & {} \frac{ C_1 }{1-C_2} (1-e^{-eV/k_BT}), \end{aligned}$$

or more explicitly;

14 $$\begin{aligned} J\simeq & {} \frac{ 32\pi m e k_B^2T^2\phi _0^{1/2} }{2\phi _0^{1/2}h^3 - h^3 k_BT A }e^{-A\phi _0^{1/2}} (1-e^{-eV/k_BT}). \end{aligned}$$

### Varying the boson density by adding the occupation number

In the model presented so far, we have ignored the occupation number part of the BE distribution, normally assumed without any limit. However, for the superconductor case, out of a total number of electron density $n_0$ in the material only *n* electrons per unit of volume form part of superconducting condensate. Furthermore, the number of super-electrons in the reservoirs is not fixed and strongly depends upon temperature *n*(*T*) since the Cooper pairs will increase as the temperature drops. In the present model the fraction $n(T)/n_0$ of super-electrons and their temperature dependency is taken into account by multiplying this fraction by the current density;

$$\begin{aligned} J \rightarrow \frac{n(T)}{n_0} \times J. \end{aligned}$$

The explicit temperature dependence of *n*(*T*) can be obtained from the the following empirical expression for the London penetration length $\lambda$^31,32^;

$$\begin{aligned} \lambda= & {} \frac{\lambda (0)}{\left( 1-[T/T_c]^4\right) ^{\frac{1}{2}}}=\left( \frac{m}{\mu _0 n(T) e^2}\right) ^{1/2}, \end{aligned}$$

where $\lambda (0)$ is the London penetration length at absolute zero. Isolating *n*(*T*) from the penetration depth expression gives its temperature dependence;

15 $$\begin{aligned} n(T)=\frac{m}{\lambda (0)^{2} \mu _0 e^2}\left[ 1-\left( \frac{T}{T_c}\right) ^4 \right] , \end{aligned}$$

which as expected goes to 0 at the critical temperature. This allows us to obtain the expression for current density of superconducting tunnel junctions;

16 $$\begin{aligned} J\simeq & {} \frac{ 32\pi m^{2} k_B^2T^2\phi _0^{1/2} }{n_0 \lambda (0)^{2} \mu _0 e h^{3} (2\phi _0^{1/2} - k_B T A) }e^{-A\phi _0^{1/2}} (1-e^{-eV/k_BT}) \left[1-\left(\frac{T}{T_c}\right)^4 \right]. \end{aligned}$$

**Figure 2:**
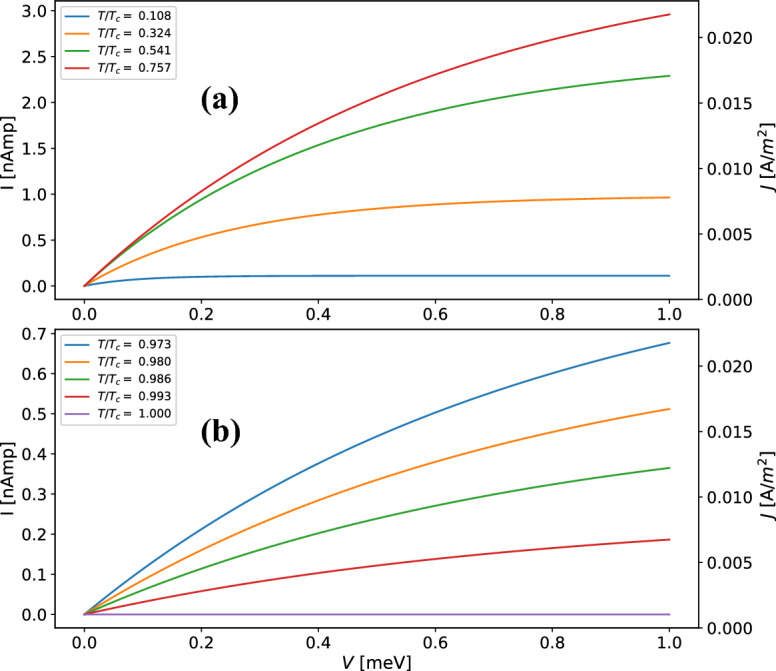
I–V characteristics obtained from Eq. (16) for Nb/Al$_{2}$O$_{3}$/Nb junctions at (**a**) low and (**b**) high temperatures bellow $T_{c}$.

In order to have a better insight of Eq. (16), we use the junction parameters for Aluminium oxide ${\mathrm {Al}}_2{\mathrm {O}}_3$ a customary barrier material obtained from previous experiments^10^. In this work, we consider tunnel junctions with barrier height $\phi =1.8$ eV, width $s =1.079$ nm and barrier cross section area A = 346 $\upmu$m $\times$ 375 $\upmu$m. Regarding superconductor and normal parameters, the values for Niobium of $\lambda (0)$ = 39 nm, $T_{c}$ = 9.25 K and $n_0$ = 8.1 $\times$ 10$^{28}$ are used. With these parameters in Eq. (16) the I–V and J–V characteristic curves are plotted in Fig. 2 for several temperatures.

**Figure 3:**
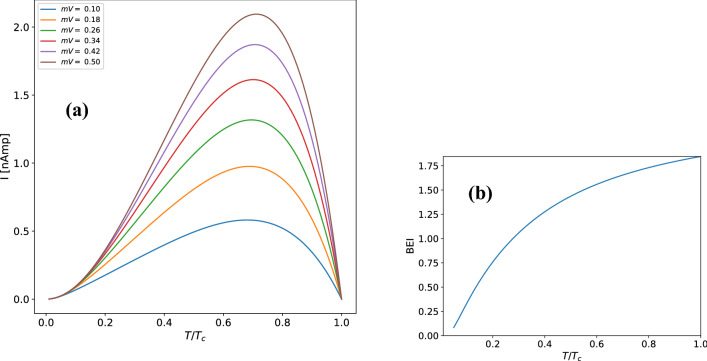
(**a**) Current versus temperature of Nb/Al$_{2}$O$_{3}$/Nb junction for a set of fixed voltages. (**b**) The Bose Einstein distribution difference integral (BEI) (Eq. 7) as function of temperature.

It is interesting to notice our analysis reveals a non-monotonic behavior of the current as a function of temperature. At low temperatures, the I–V characteristics show an increase in the values of current as temperature rises while at high temperatures there is a sharp reduction in the current values as temperature approaches to $T_{c}$. This effect can be easily observed by considering the expression of the current density for a fixed voltage and studying the current as a function of temperature as shown in Fig. 3a. Starting at zero temperature the rise in tunneling current with temperature, for temperatures T/Tc <0.7, can be understood by analyzing the integral of the Bose–Einstein distribution difference between reservoirs given in Eq. (7) (Fig. 3b). Such expression, with a logarithmic dependence, represents the net difference in the number of bosons between reservoirs at a given junction voltage and temperature. Using constant reference values of voltage *V* = 1 mV and $E_{x}=$ $1\times 10^{-4}$ eV it is possible to obtain such difference solely as function of temperature. This is shown in Fig. 3b where a significant increase between zero and transition temperature $T_c$ is observed. This behaviour indicates an important broadening of the BE distribution at finite temperatures that leads to the increase of boson occupancy probability at higher energy levels above the chemical potential and thus enhancing the transmission probability across the barrier for temperatures T/$T_c$ < 0.7.

**Figure 4:**
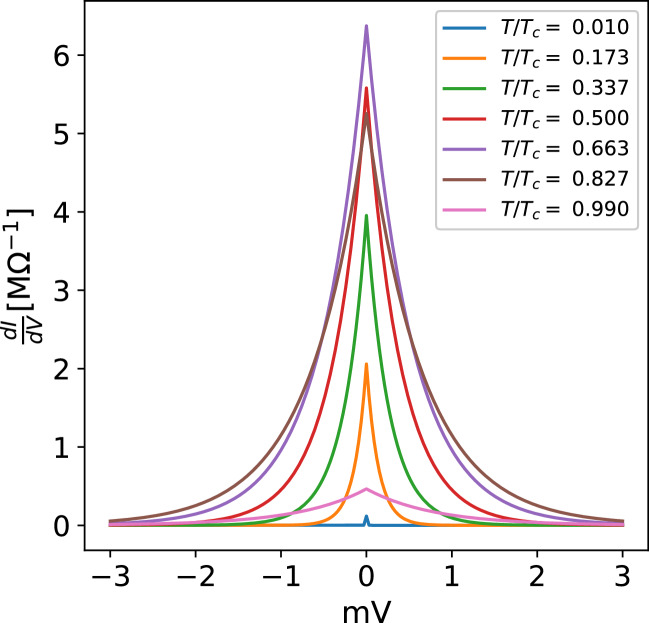
Zero bias conductance peak as function of temperature obtained from the I–V characteristics.

On the other hand, for T/T$_{c}>$0.7 the reduction of tunneling current with temperature can be explained as a consequence of the reduction in the number of Cooper pairs, expressed in Eq. (15), as the temperature approaches the phase transition. Finally, by taking the derivative of the current we obtain the conductance as a function of voltage as illustrated in Fig. 4. The conductance reveals a zero value conductance peak with the same temperature dependence as the IV characteristics described earlier. This peak could serve as an alternative explanation to a number of experimental results found in the literature to be discussed in the next section.

## Summary and discussion

An expression for tunneling of Cooper pairs that follows Bose–Einstein statistics has been obtained in Eq. (16). This equation describes the IV characteristics for S/I/S symmetric tunnel junctions as a function of temperature. For the assumption of Cooper pairs behaving as bosons to be feasible the energy from the voltage across the junction (eV) plus thermal energy ($k_{B}T$) should be kept below twice the energy gap, i.e. eV and $k_{B}T<2\Delta$. Indeed, in such regime quasiparticle excitations can not take place preventing single electrons tunneling. Given the low applied voltage restriction compared to the barrier height $\phi _{o}\gg$ eV, a constant rectangular barrier can be chosen as a good approximation for the present model. As opposed to quasiparticle tunneling, bosons tunneling show extremely low values of current density and the IV characteristics approach an asymptotic value as the voltage increases. This can be explained due to the finite number of Cooper pairs available at the superconductors which restricts the boson current. Note that for material parameters a barrier cross-section area of *A* = 346 $\upmu$m $\times$ 375 $\upmu$m has been chosen to be large, while the barrier width of *s* = 1.079 nm , rather small. These values give a current on the order of nA. This was done in order to show that although the current densities are small, this phenomenon for a large junction area and narrow barrier width should be experimentally observable. If a wider barrier is considered, the value of the current density will drop given the exponential dependence of this function in terms of the barrier width. In particular, if the size of the barrier is doubled the intensity of the current would be of order $10^{-17}$ Amperes.

The temperature dependence of tunneling current (Fig. 3a) shows a nonmonotonic behaviour with temperature. The tunneling current increases up to a maximum value around 0.7 $T/T_{c}$ as a result of the rise in tunneling probability, due to the broadening of the BE distribution with temperature. The subsequent reduction of tunneling current is due to the drop in Cooper pair density towards $T_{c}$. Finally the derivative of the I–V characteristics shows a zero bias conductance peak (Fig. 4) with its maximum around 0.7 $T/T_{c}$ that vanishes around zero or $T_{c}$.

It is interesting to notice the zero-bias conductance peak has been obtained without the need of a thin metallic layer used in the explanation of a number or previous experiments^20^. In fact, as follows from this analysis, this peak should be taken as the fingerprint of boson tunneling in other S/I/S systems where thin metallic layers can be ruled out.

## Conclusion

In previous theories of transport in S/I/S superconducting junctions, quasiparticle electron tunneling has been considered as responsible for the quantum tunneling currents, not considering the boson-like behavior of Cooper pairs. Here we proposed a simple theory for quantum tunneling of Cooper pairs that exclusively follows from their bosonic nature. It should only apply when the applied voltage and temperature are below twice the energy gap i.e. in the absence of quasiparticle excitations. Around zero bias voltage, our model predicts a zero-bias conductance peak that strongly depends on the superconductor’s temperature. This boson tunneling theory offers a possible explanation for a number of tunneling experiments where subgap currents appear that may or not include zero-bias conductance peak that varies with temperature. Furthermore, our description may shed light into experiments of Josephson junctions arrays when Bose–Einstein condensation is believed to correctly explain the observed phenomena.
