# Author Correction: Quantum tunneling theory of Cooper pairs as bosonic particles

**DOI:** 10.1038/s41598-021-96978-1

**Published:** 2021-08-30

**Authors:** Edgar J. Patiño, Daniel Lozano‑Gómez

**Affiliations:** 1 Superconductivity and Nanodevices Laboratory, Departamento de Física, Universidad de los Andes, Carrera 1 No. 18A‑12, A.A. 4976‑12340, Bogotá, Colombia; 2 School of Physical Sciences and Nanotechnology, Yachay Tech University, Urcuquí, Ecuador; 3 Department of Physics and Astronomy, University of Waterloo, Waterloo, ON N2L 3G1 Canada

Correction to: *Scientifc Reports* https://doi.org/10.1038/s41598-021-88228-1, published online 27 April 2021

The original version of this Article contained an error in Equation 16, where the term in brackets, $\left[ {1 - \left( {\frac{T}{{T_{c} }}} \right)^{4} } \right]$, was omitted.

$$ J \simeq \, \frac{{32\pi m^{2} k_{B}^{2} T^{2} \phi_{0}^{1/2} }}{{n_{0} \lambda (0)^{2} \mu_{0} eh^{3} (2\phi_{0}^{1/2} - k_{B} TA)}}e^{{ - A\phi_{0}^{1/2} }} (1 - e^{{ - eV/k_{B} T}} ). $$

now reads:

$$ J \simeq \frac{{32\pi m^{2} k_{B}^{2} T^{2} \phi_{0}^{1/2} }}{{n_{0} \lambda (0)^{2} \mu_{0} eh^{3} (2\phi_{0}^{1/2} - k_{B} TA)}}e^{{ - A\phi_{0}^{1/2} }} (1 - e^{{ - eV/k_{B} T}} )\left[ {1 - \left( {\frac{T}{{T_{c} }}} \right)^{4} } \right]. $$

The original Article has been corrected.

